# Study on γδT-Cell Degranulation at Maternal–Fetal Interface via iKIR–HLA-C Axis

**DOI:** 10.3390/cells14090649

**Published:** 2025-04-29

**Authors:** Diana Manchorova, Marina Alexandrova, Antonia Terzieva, Ivaylo Vangelov, Ljubomir Djerov, Iana Hristova, Gil Mor, Tanya Dimova

**Affiliations:** 1 Institute of Biology and Immunology of Reproduction “Acad. Kiril Bratanov”, Bulgarian Academy of Sciences, 1113 Sofia, Bulgaria; diana_man4orova@abv.bg (D.M.); marina.m.alexandrova@gmail.com (M.A.); anterzieva@abv.bg (A.T.); vangelovivaylo@gmail.com (I.V.); 2 University Obstetrics and Gynecology Hospital “Maichin Dom”, Medical University, 1431 Sofia, Bulgariaiana.hristova06@gmail.com (I.H.); 3 C.S. Mott Center for Human Growth and Development, Wayne State University, Detroit, MI 48201, USA; gmor@med.wayne.edu

**Keywords:** human pregnancy, γδT cells, inhibitory KIRs, HLA-C, degranulation

## Abstract

Maternal–fetal tolerance mechanisms are crucial during human pregnancy to prevent the immune rejection of the embryo. A well-known mechanism blocking NK-cell cytotoxicity is the interaction of their inhibitory killer-cell immunoglobulin-like receptors (iKIR) with HLA-C molecules on the target cells. In this study, we aimed to investigate the expression of iKIRs (KIR2DL1 and KIR2DL2/3) on the matched decidual and peripheral γδT cells and the localization of HLA-C ligands throughout human pregnancy. The degranulation of γδT cells of pregnant and non-pregnant women in the presence of trophoblast cells was evaluated as well. Our results showed a higher proportion of iKIR-positive γδT cells at the maternal–fetal interface early in human pregnancy compared to the paired blood of pregnant women and full-term pregnancy decidua. In accordance, HLA-C was intensively expressed by the intermediate cytotrophoblasts and decidua-invading extravillous trophoblasts (EVTs) in early but not late pregnancy. Decidual γδT cells during early pregnancy showed higher spontaneous degranulation compared to their blood pairs, but neither decidual nor peripheral γδ T cells increased their degranulation in the presence of Sw71 EVT-like cells. The latter were unable to suppress the higher cytotoxicity of γδT cells, suggesting a complex regulatory landscape beyond NK-like activity inhibition.

## 1. Introduction

Pregnancy presents an immunological paradox where the maternal immune system needs to tolerate the semi-allogeneic fetus while maintaining immune surveillance and defense against trans-placental pathogens. The lack of immune rejection of a genetically different embryo suggests the existence of mechanisms for establishing immune tolerance. In early pregnancy, the maternal–fetal interface (MFI) is abundantly populated by cytotoxic cells such as NK cells, CD8 T cells, and γδT cells [1,2]. Cytotoxic mechanisms have an important role at the maternal–fetal interface by protecting the placenta against pathogens, controlling trophoblast invasion, and eliminating fetus-reactive T cells [3]. As a dominant population, decidual NK (dNK) cells have been extensively investigated, and their cytotoxicity has been found to be tightly regulated by inhibitory and activating killer-cell immunoglobulin-like receptors (KIRs) on their surface [4,5]. These receptors interact with HLA class I molecules (HLA-G and HLA-C) expressed on invading extravillous trophoblasts (EVTs) [6,7,8,9]. KIRs specifically recognize allelic forms of HLA class I molecules, and when inhibitory KIR receptors (iKIRs) bind to classical HLA class I molecules, they trigger a signaling pathway in NK cells that blocks activation signals [10]. This interaction is crucial in controlling the cytotoxicity of dNK cells and in establishing immune tolerance toward the semi-allogenic embryo [6,9,10]. γδT cells are a unique subset of lymphocytes that express T-cell receptors (TCRs) composed of γ and δ chains. They are widely distributed in the mucosal tissues and represent a relatively small percentage (5–10%) of human circulating T lymphocytes [11,12]. In addition to TCR, γδT cells share many NK cell receptors, including KIRs, placing these unconventional T cells at the border between innate and adaptive immunity, with an important role in health and disease [13,14]. Our previous research showed that the MFI is populated by activated and proinflammatory end-stage γδT-cell effectors with a diverse TCR repertoire and strong cytotoxic potential [15]. We and others have found that decidual γδT cells produce the full range of cytotoxic molecules: perforin, granzyme A, granzyme B, granulysin, and Fas ligands [15,16,17]. Compared to the periphery, decidual γδT cells produce high levels of granzymes (A and B) and granulysin, indicating potential cytotoxicity, and lower levels of perforin, suggesting impaired cytotoxicity [15]. Since γδT cells in decidua have a different phenotype from their blood counterparts during the early stages of pregnancy, we assume differential expression of iKIRs, dependent on γδT-cell localization and/or the stage of pregnancy. In this study, we aimed to investigate the expression of iKIRs (KIR2DL1 and KIR2DL2/3) on decidual and peripheral γδT cells throughout human pregnancy and the in situ presence of their cognate ligand, HLA-C, on different subpopulations of trophoblast cells. The immediate cytotoxicity of decidual and peripheral blood γδT cells in the presence/absence of EVT-like Sw71 cells was evaluated as well.

## 2. Materials and Methods

### 2.1. Study Populations and Sample Collection

A total of 56 healthy women were enrolled in this study: 22 in early pregnancy (6–12 weeks), 20 in full-term pregnancy (38–40 weeks), and 14 non-pregnant women. Pregnancies with complications such as infection, steroid treatment, AIDS, alcohol abuse, drug abuse, and immune-related diseases were excluded. The study was conducted following the Declaration of Helsinki and approved by the Human Research Ethics Committee at the University Obstetrics and Gynecology Hospital “Maichin Dom”, Sofia, Bulgaria (No. 250569/2018). All participants provided written informed consent for the use of blood and tissue samples. Paired samples of blood and decidua and chorion were collected from women in early pregnancy, decidual and chorion samples were collected from women in full-term pregnancy, and blood samples were collected from non-pregnant women. The samples were processed promptly after collection. Blood from volunteers was collected, and five separate pools were prepared to be used in degranulation experiments.

### 2.2. Histology and Immunohistochemistry

Tissue samples (chorion and decidua) were dissected into 10 mm × 10 mm × 10 mm pieces and fixed in either formalin-free HOPE fixative (HOPE^®^ I HL001R2500, HOPE^®^ II L002C001, Innovative Diagnostik-System, Hamburg, Germany) or Bouin’s fixative. Following standard protocols for each fixative, tissues were processed and embedded in paraffin wax. Five micrometer sections were obtained and stained with H/E for histological analysis. Selected slides were stained for HLA-C or HLA-G using a three-step biotin-streptavidin enzyme method and an UltraTec Anti-Polyvalent visualization system (AFK600, SkyTec Laboratories, Logan, UT, USA). Briefly, the dewaxed (in xylene, 2 times for 10 min in each) and rehydrated (in a decreased concentration of ethanol, 5 min in each), slides were washed and treated with 3% hydrogen peroxide (in distilled water for 30 min at 37 °C) to exhaust the endogenous peroxidase. Nonspecific binding was blocked with Super block (AAA-015, ScyTek Laboratories, Logan, UT, USA) according to the manufacturer’s instructions. Then, the slides were incubated with primary rabbit polyclonal anti-HLA-C (E-AB-17922, Elabscience, Houston, TX, USA) or anti-HLA-G (E-AB-18031, Elabscience) antibody diluted in 1% BSA overnight at 4 °C in a humidified chamber. The endogenous biotin was blocked using a Biotin Blocking kit (BBK120, Scy Tek, Logan, UT, USA) according to the manufacturer’s instructions. Between the incubations, the sections were washed 3 times in PBS for 5 min. The signal was developed using 3,3-diaminobenzidine tetrahydrochloride by incubating twice for 5 min. The nuclei were lightly stained with hematoxylin. The slides were dehydrated in graded alcohol series (70%, 80%, 96%, and 100% for 5 min in each), immersed in xylene (2 times for 10 min in each), and embedded in Canada balsam (6965, Merck, Darmstadt, Germany). As a negative control, the primary or secondary antibody or HRP was omitted. For validation of the HLA-C staining specificity, tissue sections from human breast cancer were processed and stained for HLA-C in the same manner and used as a positive control [18]. The slides were analyzed using an Olympus BX51 microscope equipped with a 5.1 MP Olympus C5050Z camera (Olympus Optical Co., Ltd., Tokyo, Japan).

### 2.3. Isolation of Peripheral Blood Cells (PBMCs) and Decidual Mononuclear Cells (DMCs)

Blood samples collected in heparin-coated vacutainer tubes (BD Biosciences, Franklin Lakes, NJ, USA) and diluted twice with PBS were subjected to PBMC isolation using Lymphoprep (density: 1.077 g/mL; Axis-Shield PoCAS, Oslo, Norway). After centrifugation for 20 min at 800× *g* (brake off), the PBMC band was collected, washed with PBS, assessed for viability using a trypan blue exclusion test, and used immediately for FACS staining or for the degranulation assay. Early-pregnancy decidual tissue was obtained from the decidua basalis, and full-term decidua samples were obtained from the basal plate of the placenta. Tissue samples were thoroughly washed in PBS to remove blood clots and processed using an established protocol [16]. After mechanical disintegration of the tissue, the DMC suspension collected in sterile PBS (50 mL) was sequentially filtered through a 100 μm metal sieve and a 60 μm strainer, then centrifuged at 370× *g* for 15 min. The pellet was resuspended in sterile PBS, layered on Lymphoprep, and centrifuged at 800× *g* for 20 min without breaking. The DMC was collected from the interface, washed, assessed for viability by trypan blue test, and stained for FACS or used for the degranulation assay.

### 2.4. Cell Line, Culture Media, and Supplements

The Sw71 cell line (Cellosaurus Swan 71 CVCL_D855) is an immortalized trophoblast cell line isolated from a 7-week-old healthy human placenta [19]. Sw71 cells have an EVT-like phenotype [20]. Sw71 cells were propagated using DMEM-F12 medium (D8062) supplemented with 10% heat-inactivated fetal calf serum (FCS, 11573397), 10 mmol/L, HEPES (15630-080), 0.1 mmol/L MEM (11140-050), 1 mmol/L sodium pyruvate (11360-070), and 100 U/mL penicillin/streptomycin (15140-122), all from Gibco. PBMC and DMC were cultured in RPMI 1640 medium (R8758, Sigma Aldrich, St. Louis, MO, USA) supplemented with 10% FCS, 10 mmol/L HEPES, 0.1 mmol/L MEM non-essential amino acids, 1 mmol/L sodium pyruvate, and 100 U/mL penicillin/streptomycin. All cell cultures were maintained in a humidified incubator at 37 °C with 5% CO_2_.

### 2.5. Immunocytochemistry

Sw71 cells were cultured for 24–48 h in sterile culture chambers on a glass slide (354108, Falcon, Corning, NY, USA). The cells were fixed in 2% paraformaldehyde (PFA) in PBS for 2 h at room temperature (RT) and stained for HLA-C or HL-G using an indirect immunofluorescent method. After washing with PBS, the cells were incubated with Super block (to block the non-specific binding). For HLA-C staining, the primary purified polyclonal rabbit anti-human HLA-C antibody (E-AB-17922, Elabscience) was applied, followed by AF488-conjugated goat anti-rabbit antibody (E-AB-1055, Elabscience). For HLA-G staining, the cells were incubated with purified rabbit anti-human HLA-G antibody (E-AB-18031, Elabscience), then with goat anti-rabbit IgG (E-AB-1055, Elabscience). The negative controls were prepared by omitting primary antibodies. The slides were imaged with an ECHO Revolve microscope (RVL-100-M, Echo, San Diego, CA, USA).

### 2.6. FACS Staining and Analysis

Flow cytometry was employed to analyze the phenotypes of PBMC, DMC, and the Sw71 cell line. Cells adjusted to a concentration of 1 × 10^6^ cells per sample were subjected to immunofluorescent staining. Specific monoclonal antibodies in appropriate combinations were used, including CD3-APC (clone UCHT-1, BD Pharmingen, Franklin Lakes, NJ, USA), γδ TCR-PE (clone 11F2; BD Pharmingen), TCRγδ-FITC (clone 11F2, BD Pharmingen), CD107a-PE antibody (clone H4A3, BD Pharmingen or Biolegend), CD158a-FITC (clone HP3-E4, BD Pharmingen), and CD158b-FITC (clone CH-L, BD Pharmingen). Following incubation at 4 °C in the dark for 20 min, the cells were washed with ice-cold FACS buffer (PBS with 0.1% BSA) and fixed in 0.5% PFA in PBS. The HLA phenotyping of Sw71 cells was performed by intracellular staining for HLA-C and HLA-G. After initial washing with FACS buffer, Sw71 cells were permeabilized with a Cytofix/Cytoperm kit (00-5123, 00-5223, Thermo Fisher Scientific, Waltham, MA, USA), washed with 1× permeabilization buffer (00-8333, Thermo Fisher Scientific), and incubated with HLA-C-PE antibody (clone C-8, Santa Cruz Biotechnology, Dallas, TX, USA) and HLA-G-FITC (MEM-G/9, Exbio, Ovesna, Check Republique) for 1 h. The cells were additionally washed with 1× permeabilization buffer and FACS buffer and fixed in 0.5% PFA/PBS. Unstained cells and single-stained controls were included for gating and compensation. The doublets were excluded in the analysis. Since the stimulation could lead to higher mortality in FACS analysis, we excluded dead cells based on the unique scatter properties of viable and apoptotic cells. Apoptotic cells have high-SSC/low-FSC properties, and viable cells have high-FSC/low-SSC properties [21]. To identify and gate the population of interest (CD107+, CD158a+, or CD158b+ cells) and set the upper boundary for the background signal, we applied fluorescence-minus-one (FMO) controls or isotype control. Flow cytometry analyses were performed on a FACS Calibur BD™, and data were processed using FlowJo software v.10 (Treestar, San Carlos, CA, USA).

### 2.7. Activation of PBMCs and T-Cell Stimulation

PBMCs were stimulated in three different ways: IL-2-induced stimulation; phorbol 12-myristate, 13-acetate, and ionomycin (PMA/Ion) polyclonal activation; and TCR-cell stimulation. To ensure adequate PBMCs for all types of stimulation in each experiment and to reduce inter-assay variation, we pooled the isolated PBMCs from 5 healthy donors. We performed three independent experiments. To obtain lymphokine-activated lymphocytes (LAKs), IL-2 (100 IU/mL) was added to PBMC (1 × 10^6^ cells) suspensions for 48 h. For PMA/Ion activation, 10 ng/mL PMA (P1585, Sigma-Aldrich, St. Louis, MO, USA) and 2 uM Ionomycin (I0634, Sigma-Aldrich) were added to 1 × 10^6^ PBMCs for 6 h. For TCR cell stimulation, 1–2 × 10^6^ PBMCs were cultured in wells coated with anti-CD3 (OKT3) antibody (16-0037-85, Invitrogen, eBioscience, Waltham, MA, USA), 1 uL/mL anti-CD28 antibody (16-0289-81, Invitrogen eBioscience™, clone CD28.2), and 100 IU/mL IL-2 (10799068001, Roche, Mannheim, Germany) in the culture for 72 h. After that, PBMCs were transferred into uncoated wells for another 48 h (resting). It is important to note that after stimulation, the cells were stained with Trypan blue to assess their viability. As unstimulated PBMCs, we used a pool from three healthy donors cultured overnight in RPMI complete medium at 37 °C/5% CO_2_.

### 2.8. Degranulation Assay

The degranulation assay was based on CD107a expression detected by FACS. There are specific issues with examination of CD107a expression that must be considered during the assay setup. Because of the active and fast internalization of the CD107 molecule, it is necessary to label the immune cells with antibodies to that molecule during a period of 6 h [22]. In the current study, we conducted two sets of degranulation experiments: (1) degranulation of pooled PBMCs and (2) degranulation of PBMC/DMC samples from pregnant/non-pregnant women. We performed the degranulation assay with pooled PBMCs stimulated in different ways (as mentioned above) and with isolated, paired PBMCs and DMCs from pregnant women and PBMCs from non-pregnant women. Note that PBMC and DMC samples were not pooled. For the pooled, LAK-activated PBMCs, the degranulation analysis was performed after 48 h of stimulation. For the pooled TCR-activated PBMCs, the degranulation analysis was performed after their TCR stimulation (72 h activation and 48 h rest). For PMA/Ion-activated, pooled PBMCs the degranulation assay was conducted after their 4 h activation. Unstimulated, pooled PBMCs were subjected to degranulation after overnight culturing without any stimulus. The viability of cell suspensions was monitored using Trypan blue staining before the degranulation assay to ensure that more than 80% of the cells were vital. The degranulation assay for single PBMC and DMC samples from pregnant women and PBMCs from non-pregnant women was performed after overnight resting. The degranulation of 1 × 10^6^ pooled stimulated or unstimulated PBMCs was assessed after 6 h of incubation in RPMI complete with 5 μL of anti-CD107a-PE antibody (328608, Biolegend, clone H4A3) and 0.7 μg Monensin (M5273, Sigma Aldrich, St. Louis, MO, USA) at 37 °C/5% CO_2_, following the classical protocol for degranulation assays [22]. Three independent experiments were performed. The degranulation assays for isolated PBMCs and DMCs (from pregnant/non-pregnant women) were conducted as follows: 1 × 10^6^ PBMCs or DMCs from women in early pregnancy or PBMCs from non-pregnant women were co-cultured or not with Sw71 trophoblast cells in a ratio of 1:1 for 6 h in 1 mL medium (50% DMEM/F12 complete and 50% RPMI complete) supplemented with 0.7 μg Monensin and 5 μL of anti-CD107a-PE (555801, BD Pharmingen, clone H4A3) antibody [22]. After incubation, the cells were stained with antibodies against TCRγδ-FITC and CD3-APC (or CD56-APC) for 20 min at 4 °C, washed with 2 mL PBS/0.1% BSA, and fixed. Although we used the same clone, there was a small difference in the pattern of CD107 staining by the two used antibodies, and we definitely prefer the CD107a antibody from BD Pharmingen.

### 2.9. Statistical Analysis

Statistical analysis was performed using GraphPad Prism version 9.0 for Windows. A Mann–Whitney U test was used to compare the distribution of KIR2DL1+ and KIR2DL2/3+ γδ T cells between different groups of women (e.g., pregnant vs. non-pregnant and early pregnancy vs. late pregnancy). A Wilcoxon matched-pairs test was used to compare the proportions of KIR2DL1+ and KIR2DL2/3+ γδ T cells between paired samples (e.g., decidual vs. peripheral blood γδ T cells from the same pregnant woman). A one-way ANOVA test (Kruskal–Wallis test) was used to compare the degranulation of γδT cells. Data are presented as mean ± SEM. Statistical significance was defined as * *p* < 0.05, ** *p* < 0.01, and *** *p* < 0.001.

## 3. Results

### 3.1. HLA-C Expression by Trophoblasts Throughout Human Pregnancy

**Figure 1:**
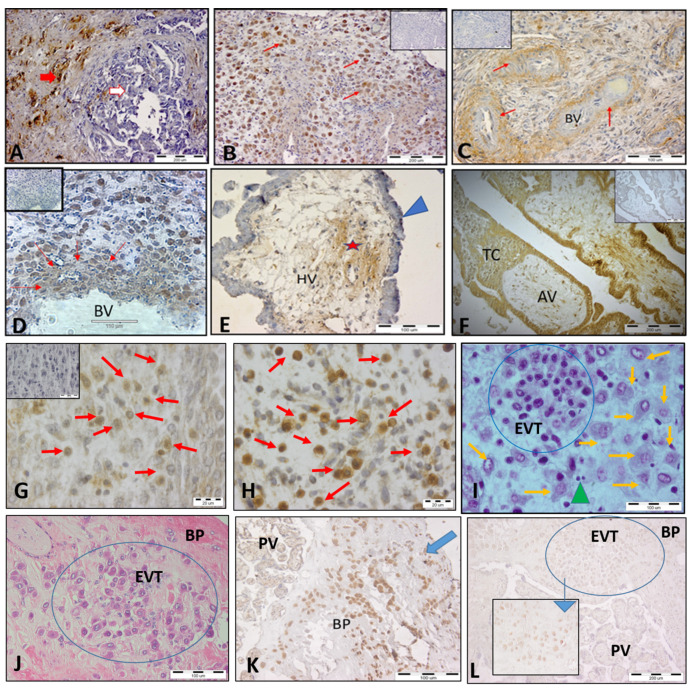
Expression of HLA-C in trophoblast populations at the MFI during human pregnancy shown by immunohistochemical staining. (**A**) Breast cancer tissue as a positive control for the specificity of HLA-C staining; tumor cells (red arrow) specifically express HLA-C but not normal cells (white arrow). (**B**) Invasion of iEVT cells into the decidua, positive for HLA-C (red arrows, 10 gw). (**C**) Weak HLA-C staining in blood vessel walls (red arrows). (**D**) HLA-C+ EVT (brown color, red arrows) remodeling the wall of the blood vessel (11 gw). (**E**) The syncytiotrophoblast of the floating villi were HLA-C-negative (blue arrowhead), and a weak positive reaction was detected in the mesenchyme (star). (**F**) HLA-C-positive intermediate cytotrophoblast cells in a trophoblastic column of an anchoring chorionic villus. (**G**) HLA-C+ iEVT cells (brown, red arrows) in the decidua (7 gw) and parallel staining for HLA-G+ (brown cells, red arrows) of the same decidua sample (**H**). (**I**) X/E staining of iEVT cells (blue circle) showing distinct morphology as compared to DSCs (orange arrows) and lymphocytes (green arrowhead). (**J**) X/E staining of EVT clusters (blue circle) in the basal plate of a full-term placenta strongly positive for HLA-G. (**K**), blue arrow) but with very weak to negative signals for HLA-C ((**L**), blue circle). The inserted small picture depicts weakly HLA-C-positive EVT cells at high magnification (scale bar 100 µm). The small pictures in (**B**–**D**,**F**,**G**) are negative controls (first antibody omitted). BV, blood vessels; HV, chorionic villi; TC, trophoblast column; AV, anchoring villi; BP, basal plate; PV, placental villi, EVT, extravillous trophoblasts.

We investigated spatial and temporal HLA-C expression at the MFI of healthy pregnant women with a focus on trophoblast subpopulations (Figure 1B–L). Breast cancer tissue was used as a positive control for the specificity of HLA-C staining, since the tumor cells specifically expressed HLA-C (Figure 1A). Our results demonstrated that HLA-C is differentially expressed in distinct trophoblast cell populations. An intense HLA-C signal was found on the interstitial EVT (iEVT) cells invading the decidua (Figure 1B,G) and on intravascular EVT (ivEVT), remodeling the blood vessels (Figure 1D). Note the numerous EVTs invading the decidua during the first EVT invasion wave (8–10 gw). A relatively weak HLA-C expression was detected in the wall of the blood vessels (Figure 1C), in the mesenchyme core of the chorionic villi (Figure 1E), and on the highly proliferative intermediate cytotrophoblast (iCT) cells in the trophoblast columns of the anchoring villi (Figure 1F). HLA-C was notably absent from syncytiotrophoblast cells of the floating chorionic villi (Figure 1E). Parallel staining for HLA-G confirmed the identity of EVT cells (Figure 1H). iEVT cells were stained for HLA-C and HLA-G on serial sections (brown cells, Figure 1G and Figure 1H, respectively). Although the decidual stromal cells (DSCs) showed weak HLA-C signals, they could be distinguished from the EVT cells by their size and morphology (Figure 1G,I). Interestingly, the stationary EVT cells in the placental bed at the end of pregnancy (Figure 1J) exhibited strong HLA-G signals but weak to negative HLA-C signals (Figure 1K and Figure 1L, respectively).

### 3.2. Decidua-Specific Higher Number of γδT+ iKIR+ Cells in Early Human Pregnancy

**Figure 2:**
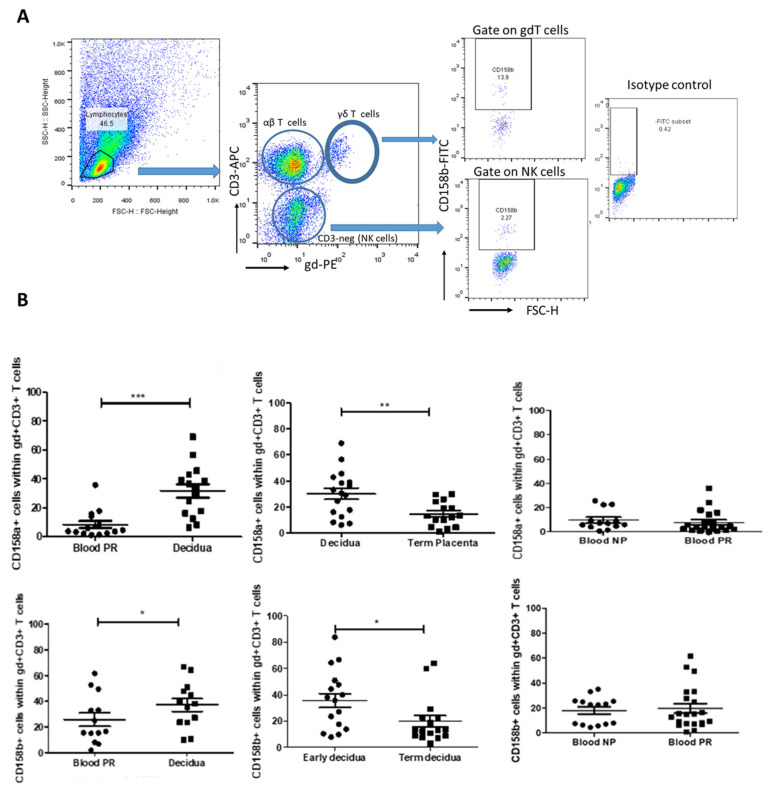
Proportion of γδT cells bearing inhibitory receptors KIR2DL1 and KIR2DL2/L3 during human pregnancy. (**A**) Representative FACS plots showing the gate strategy for iKIRs (CD158a and CD158b) detection in γδT cells. By gating on the lymphocytes and plotting CD3 vs. TCRγδ staining, we could define αβT cells (CD3+ γδ-), γδT cells (CD3+ γδ+), and NK cells (CD3-negative). (**B**) Upper row: iKIR2DL1 (CD158a) expression: left—higher proportion of KIR2DL1+ γδT cells at the MFI in comparison to their blood counterparts (paired samples, *n* = 17); middle—a decrease in KIR2DL1+ γδT cells as pregnancy progresses (*n* = 15); right—comparable levels of KIR2DL1+ γδ T cells in blood of pregnant and non-pregnant women (*n* = 20 and *n* = 13, respectively). Bottom row: iKIR2DL2/3 (CD158b) expression: left—higher number of decidual KIR2DL2/L3+ γδ+ T cells (*n* = 17) compared to their blood pairs and to those in decidua at the end of pregnancy (*n* = 16, middle); right—comparable number of KIR2DL2/L3-positive γδT cells in the blood of pregnant (*n* = 21) and non-pregnant women (*n* = 14). Statistical analysis: Mann–Whitney U test for independent groups and Wilcoxon matched-pairs test for paired groups. Data were calculated as mean ± SEM using GraphPad Prism v.5. * *p* < 0.05; ** *p* < 0.005; *** *p* < 0.001.

HLA-C is the only polymorphic MHC molecule expressed by trophoblasts and is the major ligand for the members of the KIR family [23]. We assumed that the intensive expression of HLA-C molecules at the MFI could induce the expression of iKIRs in decidual cytotoxic γδT cells. Thus, we characterized γδT cells expressing inhibitory KIRs (CD158a and CD158b) at the MFI by ex vivo quantitative analysis of these cells in a comparative manner. The proportion of decidual γδT cells bearing iKIR receptors during early pregnancy was compared to that of their blood counterparts (paired samples) and to that of decidual γδT cells at the end of pregnancy. The proportion of peripheral blood γδT cells expressing iKIRs was compared between pregnant and non-pregnant women. We found four times more γδT cells positive for KIR2DL1 at the MFI in early pregnancy in comparison to their peripheral counterparts (30.23 ± 4.3% vs. 7.84 ± 2%, *p* < 0.001, Figure 2B, upper, left). As pregnancy advanced, decidual γδ+ KIR2DL1+ cells decreased twice (14.75 ± 2.5%, *p* < 0.01, Figure 2B, upper, middle). Peripheral blood γδT+ KIR2DL1+ cells were in comparable numbers in pregnant and non-pregnant women (7.84 ± 2% and 9.67 ± 2.3%, respectively; *p* > 0.5, Figure 2B, upper, right). KIR2DL2/L3 (CD158b) was expressed by 36% of γδT cells at the MFI, following the expression pattern of KIR2DL1. The comparison of the paired decidua and blood samples from the pregnant women revealed that γδ+ KIR2DL2/L3+ cells were present in higher amounts at the MFI compared to those in the blood (38.36 ± 3.6 vs. 26.64 ± 2.4%, *p* < 0.05, Figure 2B, bottom, left). At the end of pregnancy, the number of decidual γδ+ KIR2DL2/L3+ cells reliably decreased (19.78 ± 5.7%, *p* < 0.05, Figure 2B, bottom, middle). Similar to KIR2DL1, there was no difference in the amount of KIR2DL2/L3-positive γδT cells in the blood between pregnant (19.76 ± 3.8%) and non-pregnant (18.02 ± 2.9%) women (*p* > 0.05, Figure 2B, bottom, right).

### 3.3. Spontaneous and Stimulated Degranulation of Peripheral Blood γδT Cells

**Figure 3:**
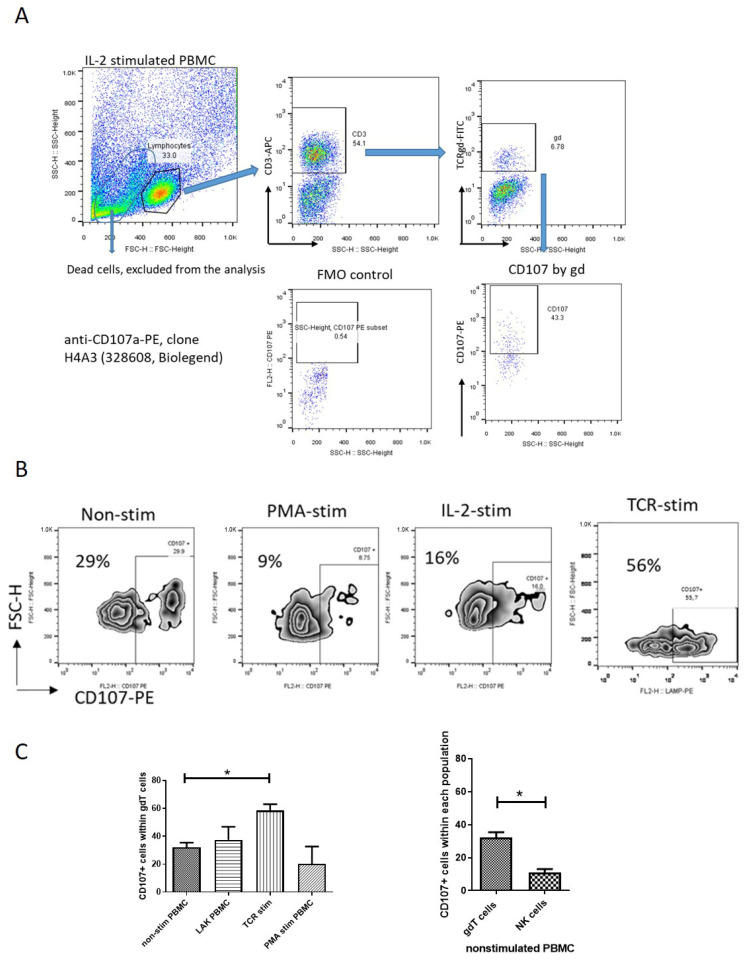
Degranulation of peripheral blood γδT cells (spontaneous and activated) based on CD107 expression. (**A**) Representative FACS plots of the gate strategy for detection of CD107-positive γδT cells. By gating on the lymphocytes and staining for CD3, we defined T lymphocytes and NK cells (CD3-negative). By gating on T cells and staining for TCRγδ, we could define γδT cells (CD3+ γδ+). CD107 expression was analyzed within γδT cells by using anti-CD107a-PE, clone H4A3 (328608, Biolegend). (**B**) Representative flow cytometry plots showing CD107a+ γδ T cells in unstimulated and stimulated pooled PBMCs. The gating on positive CD107+ cells was adjusted according to unstimulated PBMCs. (**C**) **Left**—comparison of CD107a+ γδT-cell frequency in unstimulated, IL-2-treated, PMA-stimulated, and TCR-stimulated pooled PBMCs from healthy donors (*n* = 3–5). (**C**) **Right**—spontaneous degranulation of γδT cells relative to NK cells. One-way ANOVA tests were used to compare two or more groups. Graphs display mean + SEM, analyzed by GraphPad Prism v.5. * *p* < 0.05.

Since cytotoxicity is tightly controlled in terms of cytotoxic granule polarization and degranulation, it is important to emphasize that cytotoxic potential does not necessarily mean cytotoxicity. Unlike numerous data about spontaneous degranulation of NK cells [24,25,26,27], there have been no reports about γδT-cell degranulation under steady-state conditions. We assessed γδT-cell degranulation based on CD107 expression in pooled PBMC derived from healthy volunteers. CD107a is a molecule exported to the surface of cytotoxic cells upon cell degranulation, and increased expression of CD107a reflects the killing capability of γδT cells [28]. Our gating strategy for identifying γδ T cells and quantifying CD107a expression is depicted in Figure 3A. We observed a significant level of spontaneous degranulation in γδT cells within unstimulated PBMCs from healthy donors, i.e., 32.2 ± 3.2% of γδT cells (of CD3+ cells) expressing CD107a (Figure 3C-left). Note that spontaneous γδT-cell degranulation was approximately three times higher than that observed in NK cells defined as CD3-negative cells (Figure 3C-right). To determine the impact of various stimuli on γδT-cell degranulation, we assessed the effects of the activation of PBMCs with IL-2 (LAK-activated cells), TCR stimulation (receptor-dependent activation), or PMA/Ion polyclonal stimulation (receptor-independent activation). Neither IL-2 (37.5 ± 9.4%) nor PMA polyclonal activation (20.3 ± 12.3%) significantly altered the spontaneous degranulation level of γδT cells (*p* > 0.05, Figure 3C-left). However, TCR stimulation led to a significant increase in γδT-cell degranulation, showing 58.5 ± 4.5% of γδT cells as positive for CD107a (*p* < 0.05, Figure 3C, left).

### 3.4. Degranulation of γδT Cells of Pregnant Women in the Presence/Absence of Sw71 EVT-like Cells

**Figure 4:**
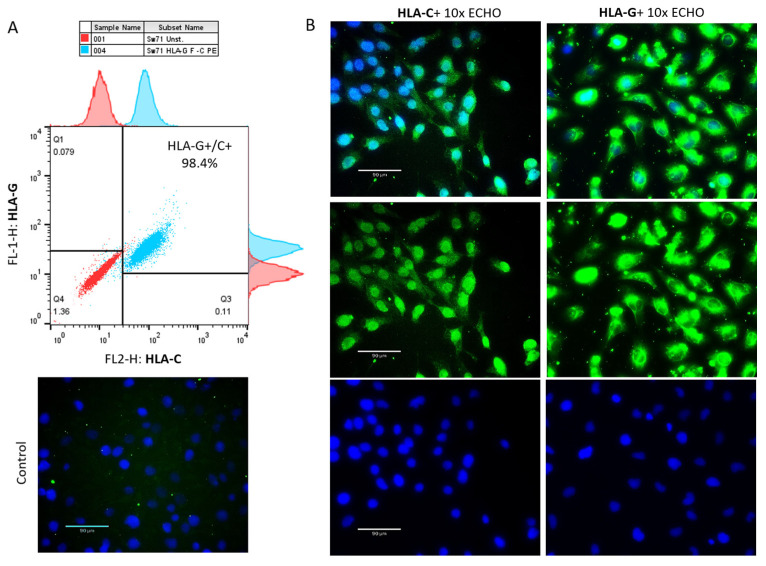
The specific HLA profile of Sw71 EVT-like cells is shown by FACS and by immunocytochemistry. (**A**) Representative FACS plot (*n* = 3) of concomitant HLA-G and HLA-C staining, demonstrating that all Sw71 cells are double-positive (HLA-G+/HLA-C+). Histograms of single HLA-G and HLA-C staining of Sw71 cells according to unstained Sw71 (up of HLA-G, right of HLA-C). (**B**) Sw71 monolayer immunostained for HLA-C (left panel) and HLA-G (right panel): a positive signal from Sw71 cells (green, middle images), Hoechst-stained nuclei (blue, bottom images), and merged images of both colors (upper images). Staining of the negative control is shown on the left side (omitted anti-HLA-C antibody). Fluorescent microscopy with 10× magnification.

**Figure 5:**
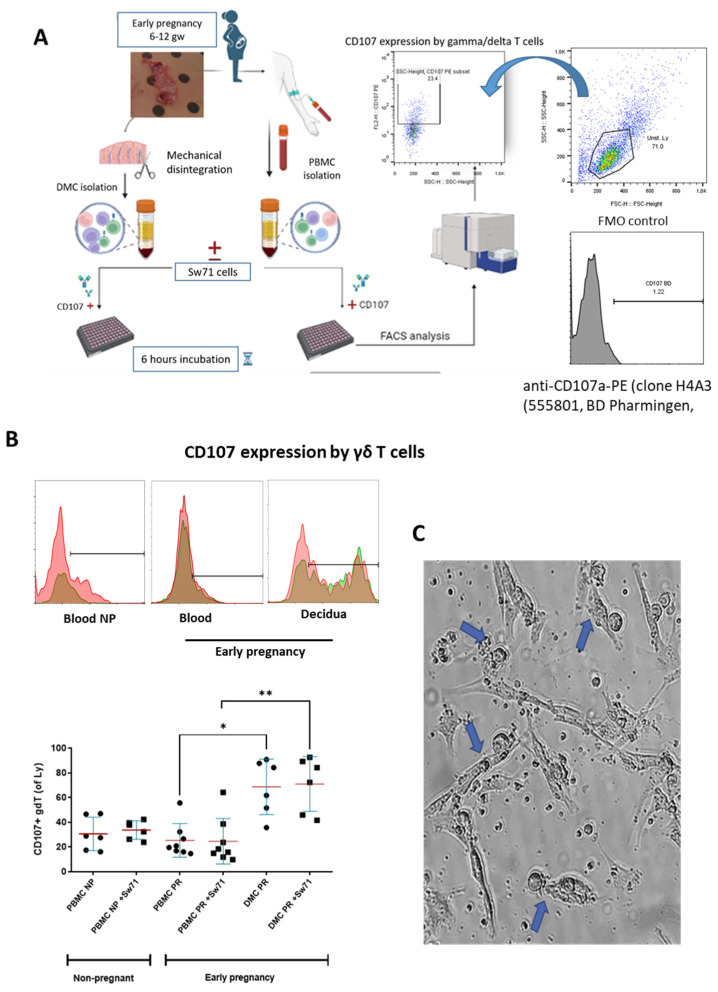
Degranulation of γδT cells from pregnant and non-pregnant women based on the measurement of CD107 expression. (**A**) Workflow of PBMC and DMC isolation, co-cultures with EVT-like Sw71 cells, degranulation assay setup, and CD107a expression using anti-CD107a-PE antibody clone H4A3 (555801, BD Pharmingen). CD107426+ cells were analyzed within the γδT-cell population, defined by TCRγδ staining of PBMCs or DMCs and gating of the lymphocytes. The FMO control was used to delineate positive cells. (**B**) **Upper row**—representative histograms showing CD107-positive γδT cells within PBMC suspensions of non-pregnant women and within matched DMC and PBMC suspensions from pregnant women (1st trimester) without Sw71 EVT-like cells (pink color) and co-cultured with Sw71 cells (green color). Both histograms (−/+ Sw71 cells) are overlaid, and the brown color represents overlapping of pink and green. (**B**) **Bottom row**—graphs showing higher spontaneous degranulation of decidual γδT cells compared to that of the matched peripheral counterparts in pregnant women (1st trimester, *n* = 8). γδT cells in the blood of pregnant (*n* = 8) and non-pregnant (*n* = 6) women degranulated to the same extent. The co-incubation of decidual or peripheral γδT cells with EVT-like Sw71 cells did not change the number of CD107+ γδT cells. Similarly, γδT cells derived from the blood of non-pregnant women did not differ in terms of their degranulation when co-cultured with Sw71 cells. Graph display mean ± SD, GraphPad Prism v.5., * *p* < 0.05, ** *p* < 0.01. (**C**) Representative image of DMCs co-cultured with Sw71 cells, showing the contacts (blue arrow) between decidual mononuclear cells and Sw71 cells during 6 h of co-incubation. No visible detrimental effect on the morphology of trophoblast cells was observed. 20× magnification.

The limited or completely blocked cytotoxicity of CTL or NK cells at the MFI is due to the presence of iKIR on the cytotoxic cells that recognize HLA molecules expressed on the fetal trophoblast [29]. We have already shown that γδT cells at the MFI have high cytotoxic potential [15] but are equipped with iKIRs recognizing HLA-C on the EVT, and thus, could be suppressed to degranulate. To check this assumption, we co-cultured γδT cells within PBMCs and DMCs isolated from pregnant women with Sw71 EVT-like cells. It was essential before co-culture to confirm the specific HLA profile of Sw71 cells. As shown in Figure 4, Sw71 cells were double-positive for HLA-G+ and HLA-C+, pointing out their EVT-like nature. The HLA profile of Sw71 cells was evaluated by FACS and immunocytochemistry. In Figure 5A, we show the workflow of peripheral blood and decidua specimens derived from women in early pregnancy and non-pregnant women to obtain PBMC and DMC suspensions. These were subjected to a degranulation assay when cultured or not with EVT-like Sw71 cells. The γδT-cell degranulation level was assessed by FACS based on CD107 expression. Our results show that the percentages of spontaneously degranulated peripheral blood γδ T cells derived from pregnant and non-pregnant women were comparable (25.30 ± 4.8% vs. 30.58 ± 5.5%, respectively; *p* > 0.05; Figure 5B) and close to the abovementioned value in the pooled PBMCs from healthy volunteers (32.2 ± 3.2%, Figure 3B). Interestingly, the decidual γδT cells of women in early pregnancy showed higher immediate cytotoxicity than their blood pairs (68.70 ± 9.2% vs. 25.30 ± 4.8%, respectively; *p* < 0.05; Figure 5B). However, the co-culture of decidual or peripheral γδT cells with Sw71 EVT-like cells did not change their degranulation. We found comparable numbers (*p* > 0.05) of CD107+ decidual γδT cells alone (68.70 ± 9.2%) and co-cultured with Sw71 cells (71.03 ± 9.1%). Similarly, the numbers of peripheral blood γδT+ CD107+ cells were comparable (*p* = 0.505) in the presence (25.30 ± 4.8%) and absence (24.64 ± 6.5%) of EVT-like Sw71 cells (Figure 5B). Also, the co-culture of Sw71 EVT-like cells with PBMCs from non-pregnant women did not significantly change the degranulation of γδT+ cells, as shown by the percentage of γδ+ CD107+ with (33.73 ± 3.05%) or without (30.58 ± 5.5%) Sw71 EVT-like cells (*p* > 0.05, Figure 5B). Although NK cells were not the focus of this study, we observed that the spontaneous degranulation of NK cells in the blood of pregnant women was lower than that of γδT cells and close to that found in pooled unstimulated PBMCs (Figure 3). Moreover, the addition of of Sw71 cells did not provoke increased NK cell degranulation (Figure S1). In Figure S1, we provide gating strategy FACS plots when staining for CD56 was applied. Usually, after 6 h incubation in the degranulation assay, we imaged the co-cultures to check if there were any changes in the forming Sw71-cell monolayer. Although we observed contacts between Sw71 cells and immune cells, no disturbance or significant changes in the morphology of Sw71 cells was observed. Thus, co-culturing of Sw71 cells with PBMC/DMC suspensions has no detrimental effect (Figure 5C).

## 4. Discussion

This study provides novel insights into the expression of iKIRs at the MFI. Here, we demonstrated, for the first time, a higher proportion of γδ T cells expressing the inhibitory KIR2DL1 and KIR2DL2/3 receptors at the MFI than in the paired blood of women in early pregnancy. Accordingly, the HLA-C molecule (KIR ligand) was well expressed by intermediate and extravillous trophoblasts. These findings suggest that the upregulation of iKIRs on decidual γδT cells during early pregnancy might correlate with high HLA-C expression at the MFI. In line with previously published data, we also found differential expression of the HLA-C molecule on trophoblast subpopulations [29,30]. HLA-C was expressed by intermediate cytotrophoblast cells in anchoring villi and on interstitial and intravascular EVTs invading deeply into the decidua. The weak or absent expression of HLA-C by the stationary EVTs in the placental bed in late pregnancy found by us and others [31] corresponds well with the decreased expression of HLA-C-specific iKIRs on γδT cells at the MFI at that time. Similar data about the predominance of iKIRs on dNK cells vs. pNK cells during early human pregnancy have been already published [32,33,34]. The authors found that the transient increase in dNK cells with KIR specific for HLA-C appears to be established by the 6th week of gestation and coincides with the peak of the decidualization of stromal cells [32]. It should be noted that studies on KIR/HLA interactions in the peri-implantation period are challenging due to the uniqueness of the HLA-C molecule and the need for appropriate models to study the early stages of implantation and placentation. The HLA-C molecule was discovered around 1970 [35] and is found only in chimpanzees, gorillas, bonobos, and humans [36]. The first article demonstrating its importance for pregnancy and placentation was published ten years after its discovery [37]. Recent studies have shown that EVTs express HLA-C in the conventionally stable β2m-associated conformational form, in contrast to the unstable “open” conformational forms expressed by somatic cells [38]. Data continue to evidence the main role of HLA-C in the regulation of NK- and CD8 T-cell activity [38,39,40,41,42]. Our study adds γδT cells to the palette of cytotoxic cells at the MFI that might recognize the HLA-C molecule. Moreover, we previously reported that the innovative HLA-C-expressing 3D Sw71 blastocyst surrogate of the peri-implantation human embryo could be a useful model for in vitro investigation of KIR/HLA interactions during implantation [20,43].

It has been shown that the affinity of iKIRs for their HLA ligands varies between pregnancies, exposing dNK cells to different levels of inhibition [44,45]. This could influence trophoblast invasion and implantation/pregnancy as a whole. Interesting data have shown that immature NK cells do not express any receptors for MHC class I, meaning that dNK cells acquire their KIR repertoire within the uterine microenvironment. Thus, factors present in the endometrium, decidua, or both may affect dNK-cell receptor acquisition [46]. The maternal HLA-C genotype has been found to be significant, as it affects the development of dNK cells through interactions with maternal KIR, known as “licensing” or “education” [47]. We observed relatively high spontaneous cytotoxicity (degranulation) of γδT cells in a steady state, supporting the hypothesis that they often have a phenotype of preactivated or ready-to-act cells in a healthy organism [15,16]. Here, we found that only TCR activation of γδT cells increased their cytotoxicity but not IL-2 or PMA activation. Early pregnancy, as a condition, also did not influence the cytotoxicity of peripheral blood γδT cells. However, γδT cells at the MFI during healthy early pregnancy showed higher spontaneous degranulation compared to their peripheral blood counterparts. Yu and colleagues found that peripheral blood γδT cells in women with recurrent spontaneous abortions (RSA patients) might have elevated cytotoxicity, pointing out their contribution to RSA [48]. These data are questionable, bearing in mind the very short incubation of PBMCs with CD107 antibody (only 30 min) performed in this study. According to the classical protocol for evaluation of cytotoxicity based on the CD107 molecule, this incubation must be 6 h [22]. It should be noted that decidual γδT cells are a heterogeneous population and probably include distinct states of functional competency. Our previous research showed that the maternal–fetal interface is populated by activated and pro-inflammatory end-stage γδT-cell effectors with a diverse TCR repertoire and strong cytotoxic potential [16]. We have shown the specific predominance of TRGV2+ perforin low/granulysin high γδ T-cell effectors with reduced levels of Th1 activity in early pregnancy decidua, suggesting a peculiar role for γδT cells at the MFI [15]. Here, we observed that more than half of decidual γδT cells degranulated spontaneously, and a similar proportion did not express iKIRs. How the degranulation level corresponds to iKIR signaling was depicted in a recently published model of NK cell development/activity [49]. According to this model, there are four distinct states of NK cells: (1) CD56bright NK cells produce mainly cytokines, (2) CD56dim NK cells negative for iKIR show upregulated cytolytic effector molecules but are unable to generate mature lytic granules, (3) CD56dim cells expressing iKIR at a low level can make cytolytic granules but have depleted cytolytic reservoirs due to more frequent degranulation, and (4) CD56dim cells with high levels of iKIR due to upregulated HLA-C have a higher threshold of activation and greater cytolytic reservoirs. Whether γδT cells, as cells expressing CD56 and sharing many NK receptors, follow the same model or not, it would be worth investigating their TCR as the main receptor. A key question with respect to γδT cell cytotoxicity in this study was whether the presence of trophoblast cells could make a difference. Co-incubation of PBMCs and DMCs from pregnant or non-pregnant women with EVT-like HLA-C+/HLA-G+ cells showed no change in degranulation levels of either decidual or peripheral γδT cells. Although we observed contacts between the immune cells and Sw71 cells, the morphology of the trophoblast cells was preserved. Indeed, the detection of immune synapses does not always mean killing. The elegant study of the Tilburgs group showed that in the immune synapses between decidual NK cells and trophoblast HLA-G was trogocytosed by NK cells to establish immune tolerance [50]. The co-culture data did not support our assumption that HLA-C interacting with iKIR+ decidual γδT cells would decrease their spontaneous degranulation. But we could also suggest that γδT cells do not kill healthy invading EVT cells, which is line with the suggested role of γδT cells in controlling EVT invasion and promoting decidualization [51]. A plausible explanation could be that the iKIR/HLA-C axis has no role in the functional competence of decidual γδT cells when the pregnancy is already established and/or the γδT cells are present at MFI mostly to fight placental infections. The higher spontaneous degranulation of the relatively small population of decidual γδT cells (vs dNK cells) is necessary to control the neoplastic transformation that could be happen in places with excessive tissue remodeling, such as the maternal–fetal interface, and to deal with numerous stressed or senescent cells. Stressed cells release small amounts of phosphoantigens that specifically activate γδT cells [52,53]. However, further research is needed to ensure direct cytotoxic cell–target cell contact using isolated γδ T cells and HLA-C-negative targets. Our findings that the presence of EVT-like Sw71 cells did not further increase the degranulation of decidual or peripheral γδ T cells correspond to previously published data about the trophoblast-killing activity of CTL and dNK cells. Tilburgs T. and colleagues conducted experiments involving a 12 h co-culture of isolated primary EVTs with decidual CD8 T cells from the same woman from another pregnancy and with peripheral CD8 T cells from an unrelated donor. Their results demonstrated that trophoblast cells did not induce degranulation of CD8 T cells [54]. Similar findings were observed for both uterine and peripheral NK cells. Despite multiple interactions between primary EVTs and dNK cells from the same pregnant woman, the dNK cells did not degranulate in the presence of EVTs. Both peripheral and decidual NK cells are unable to kill EVTs, even when activated by pro-inflammatory cytokines [55]. Moreover, while dNK cells can lyse decidual stromal cells infected with HCMV, they do not kill infected EVTs [55]. However, upon hyperstimulation with IL-2, dNK cells induced apoptosis in trophoblast cell line HTR-8/SV40neo [55]. However, other studies have reported that dNK cells can degranulate and kill trophoblast cells infected with Zika virus [56]. ZIKV infection causes endoplasmic reticulum stress in trophoblasts, leading to down-regulation of HLA-C and HLA-G [56]. A series of studies by Mikhailova et al. on the cytotoxicity of IL-2 stimulated NK cells (in PBMC suspension) against JEG-3 trophoblast cells showed that it was weaker in pregnant compared to non-pregnant women [57]. Importantly, like the other cytotoxic cells at the MFI (uNK cells and CD8 T cells), γδT cells did not show cytotoxic activity against the trophoblast cells.

## 5. Conclusions

In early human pregnancy, a higher proportion of γδ T cells at the maternal-fetal interface express inhibitory KIR2DL1 and KIR2DL2/3 receptors, coinciding with HLA-C expression on the early pregnancy intermediate and extravillous trophoblast. Decidual γδT cells degranulate more than their peripheral blood counterparts. However, HLA-C+ trophoblast cells failed to suppress γδT-cell degranulation, suggesting a more complex role for γδT cells in maternal–fetal tolerance beyond NK cell-like activity.

## Data Availability

The datasets generated and analyzed during the current study are available from the corresponding author upon reasonable request.

## Supplementary Materials

The following supporting information can be downloaded at: https://www.mdpi.com/article/10.3390/cells14090649/s1, Figure S1: Degranulation of γδT cells and NK cells in PBMCs of pregnant women, co-cultured or not with extravillous Sw71 cells. Representative FACS plots, showing CD107 staining and gate strategy for defining of γδT and NK cells (gate on lymphocytes). Note the relatively high spontaneous γδT-cell degranulation compared to that of NK cells.
