# Supplementary figures and images for: Study on γδT-Cell Degranulation at Maternal–Fetal Interface via iKIR–HLA-C Axis
Source: Cells. 2025 Apr 29;14(9):649. doi: 10.3390/cells14090649 (PMC12071288; doi:10.3390/cells14090649)

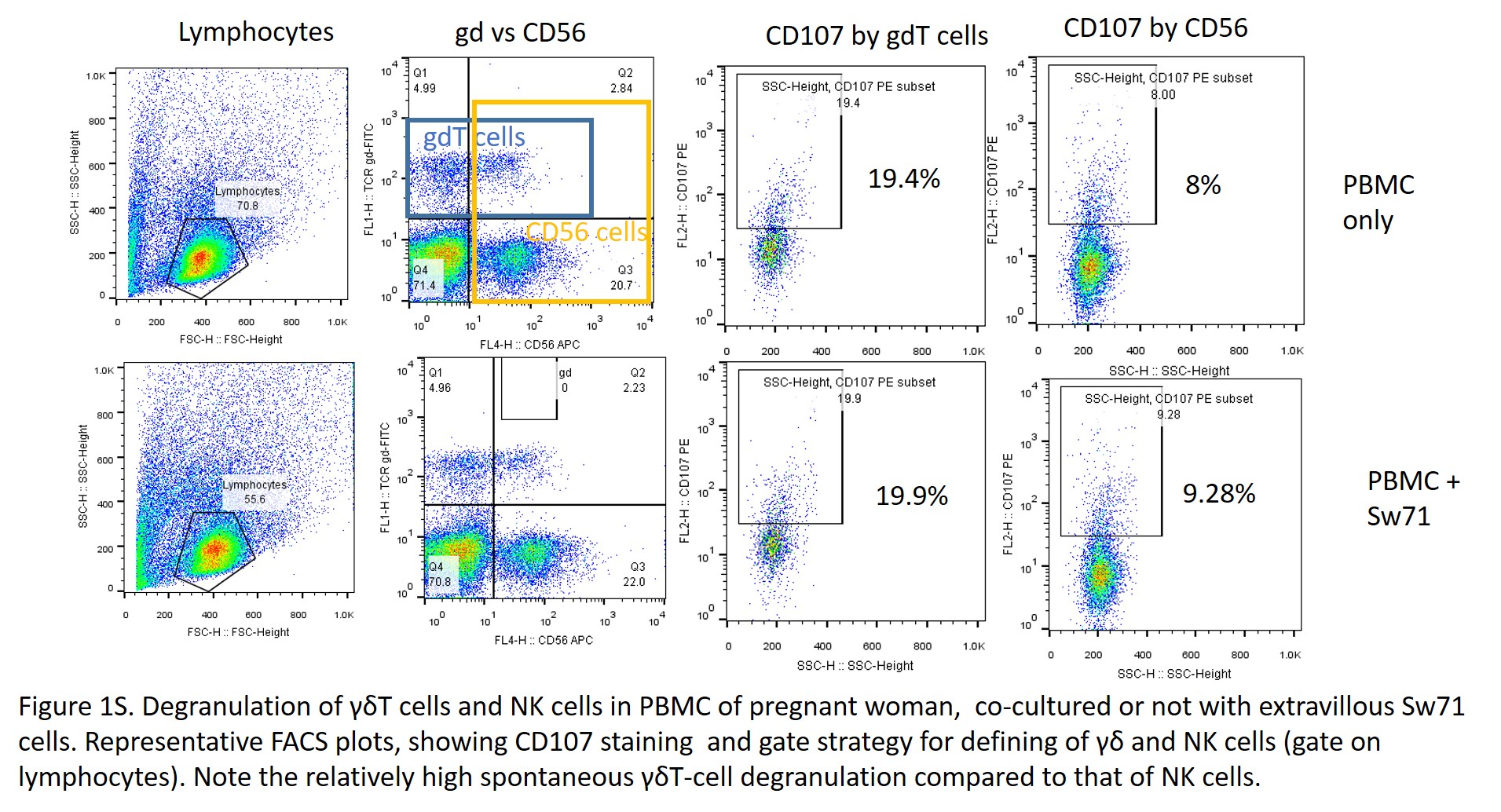

Supplement: Supplementary file 1 [file cells-14-00649-s001.zip › cells-3562677-supplementary.jpg]
